# Timeliness of the outbreak surveillance and response process: a retrospective cross-sectional multicountry assessment in the WHO African Region

**DOI:** 10.1136/bmjph-2026-005377

**Published:** 2026-11-03

**Authors:** Fleury Hybriel Moussana, Mamadou Saliou Kalifa Diallo, Kofi Emmanuel Dzotsi, Nicksy Gumede, Charles Okot, Etien Koua, Otim Ramadan, Dick Damas Chamla, Marie Roseline Darnycka Belizaire

**Affiliations:** 1 World Health Organization Regional Office for Africa, Brazzaville, Congo; 2 EPR, World Health Organization Regional Office for Africa, Brazzaville, Congo

**Keywords:** Disease Outbreaks, Public Health, Communicable Disease Control

## Abstract

**Introduction:**

Early detection and response to outbreaks are among the highest expectations of public health responsibility. However, in most WHO African countries, this commitment often encounters persistent challenges that reflect the weakness of health systems and necessitate specific analyses to learn lessons and make timely decisions. Analysing timeliness from detection to response offers a particular advantage by highlighting the different delays recorded at each stage, which guide on the identification of challenges that may arise within the system.

**Methods:**

A retrospective cross-sectional study of outbreaks reported in the WHO African Region in 2024 was conducted. This study focused on a descriptive analysis of the reported outbreaks and the computation of median time for seven key indicators associated with four core milestones: detection, notification, investigation and initiation of response.

**Results:**

Most reported outbreaks were caused by viral agent, with vaccine-preventable diseases being the most frequently reported category. Overall medians (IQR) were 4 (1–7) days for detection, 0 (0–2) days for notification, 0 (0–0) days for sample collection, 1 (0–2) days for sample reception, 2 (1–4) days for laboratory turnaround, 3 (1–8) days for laboratory confirmation and 1 (0–8) days for response initiation. Significant delays were noted for specific diseases, and at particular stages of the surveillance-to-response cascade, highlighting persistent gaps and weaknesses in outbreak detection and response systems across the Region.

**Conclusion:**

Significant delays for specific diseases were noted, emphasising bottlenecks that require strong and sustained commitment from governments and an investment in resources.

**WHAT IS ALREADY KNOWN ON THIS TOPIC**

- Timeliness is essential for effective outbreak detection and response in the WHO African Region. Previous studies have shown delays in notification, laboratory confirmation and response, especially for vaccine-preventable and viral diseases.

**WHAT THIS STUDY ADDS**

- Provides evidence on timeliness across detection, notification, investigation and response from 45 Member States of the WHO African Region; and shows that major delays persist in detection and lab processes for several high-burden diseases.

**HOW THIS STUDY MIGHT AFFECT RESEARCH, PRACTICE OR POLICY**

- Supports countries in monitoring and improving IDSR implementation. It offers a practical basis for routine timeliness assessment to inform policy and guide resource allocation.

## Introduction

 The WHO African Region is increasingly facing recurring public health emergencies. Each year, approximately 100 public health emergencies, including health humanitarian crises and epidemics, are reported by Member States.^1,2^ These emergencies are often exacerbated or prolonged due to less resilient health systems, security and economic crises, or climate-related problems.^3^ In this context, the WHO continues to support Member States in strengthening surveillance and response to improve public health protection and save lives. The implementation of the Integrated Disease Surveillance and Response (IDSR) strategy and the development of the International Health Regulations provide frameworks for this assistance to countries.

However, Member States face persistent challenges during epidemics, which are generally associated with the increase in disease cases,^4^ which can place significant pressure on hospitals, leading to their saturation, increased mortality, loss of public confidence^5^ and substantial economic impacts. In this context, managing the timeliness during the different phases of epidemics is of paramount importance. Hence, the need to conduct regular assessment of timeliness, from outbreak detection to response, involving several milestones of the surveillance and response processes. The importance of conducting such assessment lies in the fact that it provides public health authorities with critical advantage in the outbreak surveillance and response process, enabling effective planning and interventions.^1^

Despite the importance of timeliness in outbreak control, there is limited evidence describing the timeliness of key surveillance and response milestones across the WHO African Region within the IDSR framework, particularly across the full outbreak surveillance-to-response cascade. Previous assessments in the WHO African Region have primarily focused on selected outbreak milestones with limited assessment of performance against IDSR-recommended timeliness thresholds across the full surveillance-to-response cascade.^1^ This highlights the need for more comprehensive and updated evidence.

This study described the characteristics and distribution of outbreaks reported in the WHO African Region in 2024 and assessed the timeliness of four key surveillance and response milestones across the outbreak surveillance-to-response cascade. It further examined variations in timeliness by disease type and by stage of the surveillance and response process to identify bottlenecks in the disease surveillance and response process and inform targeted improvements in outbreak preparedness and response.

## Materials and methods

This was a retrospective cross-sectional descriptive analysis of outbreaks reported in the WHO African Region, which consists of 47 Member States, in 2024. The data on these outbreaks were collected during the IDSR implementation assessment conducted between March and July 2025. Data were collected using a standardised, self-administered online questionnaire, available in English and French on the Kobo Toolbox platform. The questionnaire was sent to Member States by email via WHO country offices, which coordinated its completion with the relevant departments within the Ministries of Health.

However, this approach has certain limitations. First, although a standardised questionnaire was used, slight differences in the interpretation of some questions among respondents remain possible. Second, the comprehensiveness and quality of the source data may vary from one country to another, which could influence certain indicators. Finally, there is a lack of systematic external validation, like independent audits or field assessments, of the information collected through.

To reduce the impact of these limitations, some measures were implemented both before and after data collection. An orientation webinar was organised to present the questionnaire and ensure a consistent understanding of it, while surveillance officers in WHO country offices supported Member States throughout the process. Member States were also encouraged to involve national surveillance units and to review the data before submission. Finally, a consistency check and data cleaning exercise were conducted. Member States were subsequently given the opportunity to review their data for any updates or corrections prior to analysis.

Four core milestones were used in this study to assess the surveillance and response processes for outbreaks; these include detection, notification, investigation and initiation of response. The specific aspects assessed for investigation include sample collection, sample transportation and laboratory reporting of results. Table 1 provides definitions of the time intervals for each of the milestones.

**Table 1:** Indicators for evaluating timeliness of outbreak detection, investigation and response

Milestone number	Milestone name	Indicator	Definition	Threshold	Source
1	Detection	Time to detection	Number of days from onset of index case (or occurrence of an unusual cluster at the community level) to arrival of first outbreak case at the health facility	<3 days	^28^
2	Notification	Time to notification	Number of days from initial outbreak case seen at the health facility (or date of outbreak threshold crossing at the health facility) and reporting to the district health team	Within 24 hours	^28^
3	Investigation	Time to sample collection	Number of days from detection and sample collection for laboratory testing	Within 48 hours	^29–31^
		Time to sample receipt at the testing laboratory	Number of days from sample collection to its arrival at the testing laboratory	24–48 hours	^29–31^
		Laboratory turnaround time	Number of days from sample arrival at the testing laboratory to reporting of results	Within 24 hours	^31–33^
		Time to laboratory confirmation	Number of days from sample collection to reporting of results	Within 7 days	^31^
4	Response	Time to initiation of response	Number of days from notification to initiation of concrete response nationally recommended	Within 48 hours	^28^

We included disease outbreaks occurred that were reported by 44 Member States of the WHO African Region (AFRO) that participated in the assessment, which had dates of interest available for each of analysed milestone. However, disease outbreaks occurred but not reported during the assessment of IDSR implementation were not included. It is important to note that according to WHO’s monitoring outbreak list, few outbreaks occurred in the three Member States that have not participated in the assessment.

Since the date of notification of the district was not available, we used the date of notification of the national level as a proxy to compute time to notification, and time to initiate response.

The total of 83 reported outbreaks had dates for onset of symptoms, detection and notification. And so, all these 83 outbreaks were included in the calculation of time to detection and time to notification. For time to sample collection, time to reception at the testing laboratory, laboratory turnaround time and time to laboratory confirmation, 82 were included due to the non-availability of the related dates for hand, foot and mouth. For the initiation of concrete response, 82 outbreaks were included, as there was one missing date for one measles outbreak. The coherence of these dates was reviewed, and Member States had the opportunity to review their data and make final correction before analysis.

Diseases were classified by pathogen type, and descriptive statistic was employed to describe the outbreaks that occurred. Median time and IQR were then computed for each of the milestone and disease outbreak to measure the delay. To identify steps associated with long delays, thresholds defined in table 1 were applied.

Zero day in this study means in less than 24 hours. Data cleaning and analysis were done using Excel and R software V.4.4.3.

## Results

All the results are consigned in the online supplemental table 1 and table 2.

**Table 2:** Eligible outbreaks, threshold attainment and 95% CIs

Indicator	Total outbreaks	Eligible outbreaks	Met threshold	Met threshold (%)	CI95_L	CI95_U
Time to detection (<3 days)	83	83	33	39.8	29.2	51.1
Time to notification (≤1 day)	83	83	61	73.5	62.7	82.6
Time to sample collection (≤2 days)	83	82	78	95.1	87.8	98.6
Time to sample receipt (≤2 days)	83	82	63	76.8	66.2	85.4
Laboratory turnaround time (≤1 day)	83	82	39	47.6	36.8	58.4
Time to laboratory confirmation (≤7 days)	83	82	60	73.2	63.6	82.8
Time to initiation of response (≤2 days)	83	82	48	58.5	47.9	69.2

### Description of outbreaks

In 2024, a total of 83 outbreaks caused by 21 distinct diseases were reported by 45 Member States that participated in the assessment. Most of these outbreaks were caused by infectious agents, including viral (73%), bacterial (24%) and parasitic (1%), and more than 66% from diseases for which vaccines are available. The most frequently reported outbreaks were measles (25 outbreaks—30%), mpox (14 outbreaks—17%) and cholera (13 outbreaks—16%). Outbreaks caused by these three diseases affected 170 267 people and caused 1888 deaths. These figures represent 75% of all cases and 71% of all deaths recorded across the 83 outbreaks.

### Analysis of the seven milestones and identifying of steps with long delays

For detection, reported outbreaks of bubonic plague, cholera, COVID-19, dengue, hepatitis E, chronic ulcers of unknown origin and yellow fever were detected in fewer than 3 days (table 2). Detection was late for other reported outbreaks, with extended delays observed for diphtheria (3 days—IQR=2–6) and poliomyelitis (3 days—IQR=2–18), measles (4 days—IQR=2–6) and pertussis (4 days—IQR=4–4), Lassa fever (5 days—IQR=3-6), mpox (5 days—IQR=3–14) and Rift Valley fever (5 days—IQR=3–6), malaria (6 days—IQR=6–6) and listeria (7 days—IQR=7–7). The situation was critical for influenza (8 days—IQR=8–8); meningitis (9 days—IQR=9–9); rabies (12 days—IQR=12–12); hand, foot and mouth disease (14 days—IQR=14–14); and Marburg virus disease (18 days—IQR=18–18).

Most of the reported outbreaks were notified to the next level within 1 day. Exceptions were noted for pertussis (4 days—IQR=4–4), poliomyelitis (12 days—IQR=2–43), chronic ulcers (29 days—IQR=29–29), malaria (45 days—IQR=45–45) and meningitis (103 days—IQR=103–103).

For investigation purposes, samples were collected within 24 hours from the detection for most reported outbreaks requiring laboratory testing, except for chronic ulcers of unknown origin. Samples were generally received at the testing laboratories within 2 days from the collection, but extended times were observed for diphtheria (3 days—IQR=2–11), pertussis (4 days—IQR=4–4), meningitis (8 days—IQR=8–8) and poliomyelitis (8 days—IQR=2–12). From the reception of samples, results were reported within 1 day for fewer reported outbreaks, while others experienced delays, with the notable exception observed for poliomyelitis (35 days—IQR=23–58).

Laboratory confirmation tests were done within 7 days from sample collection for many of the reported outbreaks. Long delays were observed for pertussis (8 days—IQR=8–8), diphtheria (9 days—IQR=8–14), meningitis (9 days—IQR=9–9), chronic ulcers of unknown origin (24 days—IQR=24–24) and poliomyelitis (47 days, IQR=28–74).

Time to initiation of response was within 2 days for many of the reported outbreaks. However, delayed response was observed for mpox (4 days—IQR=0–24), malaria (4 days—IQR=4–4), measles (6 days—IQR=1–11), influenza (7 days—IQR=7–7), Rift Valley fever (9 days, IQR=4–13), hepatitis (37 days) and chronic ulcers of unknown origin (122 days).

## Discussion

Our aim was to describe the characteristics and distribution of outbreaks that occurred in the WHO African Region in 2024 and assess the timeliness of four core surveillance and response milestones. Results show that outbreaks caused by diseases for which vaccines are available were frequently reported, which is consistent with the recent warning issued by WHO, UNICEF and Gavi.^6^ However, since these diseases differ in terms of vaccination strategies, the observed frequency of these outbreaks should not be interpreted solely as a reflection of routine immunisation performance. Other possible factors may include immunity gaps, inadequate implementation of preventive measures and limited adherence to public health recommendations.^6–8^ We also found that viral pathogens were more frequently reported than others, consistent with findings from previous study.^9^ The outbreak profile was dominated by measles, mpox and cholera, to which can be added dengue, poliomyelitis, and Lassa and yellow fevers. The situation is critical, particularly for poliomyelitis since it is one of the diseases targeted for eradication.

Prolonged time to detection for many disease outbreaks, including diphtheria; poliomyelitis; measles; pertussis; Lassa fever; mpox; Rift Valley fever; malaria; listeria; influenza; meningitis; rabies; hand, foot and mouth disease; and Marburg virus disease, indicates serious concern over the timeliness of identification and confirmation of signals. In the recent past, delayed detection for viral haemorrhagic fevers such as Lassa fever,^1,10^ Rift Valley fever and Marburg virus disease^1^ has been documented in the WHO African Region. This situation places significant pressure on health public authorities, since these diseases carry high risk. For diphtheria, measles, pertussis, rabies and influenza, a similar issue has been documented in the surveillance system in other regions.^11^ The failure in early detection for these diseases may stem from several factors, including weak detection capacity^12^ and persistent under-reporting in the routine surveillance data.^13^

Time to notification was inadequate for meningitis, malaria and poliomyelitis and raises serious concerns on notification systems. This result corroborates the conclusions of a systematic literature review^14^ conducted in another region, which highlighted the inability of many national notification systems to meet the deadlines specific to each disease. Several factors may explain these findings, including coordination gaps, limited data-sharing and infrastructure challenges across WHO African Region Member States;^15^ conflict, resource limitations and weak health service delivery;^16^ limited laboratory and detection capacity;^17^ and desensitisation of both population and health workers who perceive symptoms as routine in endemic settings.

On the laboratory side, the delays in testing for diphtheria, meningitis, poliomyelitis and pertussis highlighted may stem from systemic challenges. One of the critical challenges may be the inadequate diagnostic capacity in the subnational laboratories. This was documented in a recent study, indicating the lack of necessary PCR infrastructure for priority diseases in laboratories across multiple countries.^17^ This context therefore may necessitate the transport of samples to regional or reference laboratories, which may introduce delays. This was documented by WHO, particularly for diphtheria.^18^

Specifically, for poliomyelitis, delays may be attributed to testing backlogs and supply issues.^19^ Additionally, delays may occur during the laboratory confirmation and characterisation of detected polioviruses, where intratypic differentiation and genetic sequencing may be required, sometimes necessitating the shipment of isolates to specialised reference laboratories.^20^ The situation may have been exacerbated by reductions in funding for polio eradication programmes, resulting in decreased environmental surveillance, logistical challenges in sample collection and limited financial support for WHO AFRO.^21^

Response was delayed for hepatitis E, influenza, malaria, measles, mpox and Rift Valley fever. This is undoubtedly a signal of multiple challenges that the Member States from WHO African Region are facing. One of the major challenges may be the competing health emergencies associated with simultaneous outbreaks in a setting of resource constraints,^22^ which reduces therefore the capacity for rapid initial response to each emergency or outbreak. The weak public health systems may constitute another challenge for Member States. Indeed, the continent’s health systems are often under-resourced and poorly equipped, leading to delays in diagnosis and treatment, which in turn contribute to the spread of infections and elevated mortality rates.^23–25^ Likewise, the lack of trained personnel and logistical challenges in the procurement and distribution of reagents and equipment may also impede effective disease management.^25–27^

## Conclusion

This study shows that outbreaks of vaccine-preventable diseases were frequent in 2024, with viral pathogens being dominant. The analyses also revealed significant delays in the surveillance and response processes for outbreaks in 2024, particularly from detection to response initiation as recommended by national health policies. This suggests major and persistent challenges within public health systems across Member States in the region.

A key limitation of the analysis is that all the outbreaks that occurred in 2024 in the WHO African Region were not included, particularly those from the two Member States that did not participate in the assessment. Including those would have enabled a full situation of the region.

Another limitation is that multiple outbreaks within the same Member States may not be fully independent due to shared surveillance and health system characteristics.

In addition, the national notification date was used as a proxy for the district-level notification date, as the latter was unavailable. This may have affected the estimation of notification timeliness. However, based on the available national notification dates, most reported outbreaks were notified within the recommended timeframe, suggesting that the overall interpretation of notification timeliness is unlikely to be substantially affected.

Despite these limitations, the results of this study provide a basis for annual and regular monitoring of delays in the setting of IDSR. These also provide insight into the benefits that health systems can derive from addressing identified delays. Sustained strengthening of surveillance systems, including laboratory capabilities, strategic investment in human and logistical resources, and sustained political commitment constitute critical aspects which can lead to reducing delays and saving people’s lives.

## Supplementary material

10.1136/bmjph-2026-005377 online supplemental table 1

## Data Availability

Data are available upon reasonable request.
