# Supplementary material for: Timeliness of the outbreak surveillance and response process: a retrospective cross-sectional multicountry assessment in the WHO African Region
Source: BMJ Public Health. 2026 Nov 3;4(4):e005377. doi: 10.1136/bmjph-2026-005377 (PMC13735347; doi:10.1136/bmjph-2026-005377)
Supplement: Supplementary file 1 [file bmjph-4-4-s001.docx]

**Supplementary table 1**- Outbreak characteristics and timeliness (median time and interquartile range) of key surveillance and response milestones by disease*

| Disease name | # outbreaks | Cases | Deaths | Detection | Notification | Investigation^2^ | | | | Response^1,2^ |  |
| --- | --- | --- | --- | --- | --- | --- | --- | --- | --- | --- | --- |
|  |  |  |  | Time to detection | Time to notification | Time to sample collection | Time to sample receipt at the testing laboratory | Laboratory turnaround | Time to laboratory confirmation | Time to initiation of response | Pathogen type |
| Bubonic plague | 1 | 59 | 19 | 1 (1-1) | 0 (0-0) | 0 (0-0) | 1 (1-1) | 3 (3-3) | 4 (4-4) | 0 (0-0) | Bacterial |
| Cholera** | 13 | 104007 | 1482 | 1 (0-3) | 0 (0-2) | 0 (0-0) | 0 (0-1) | 2 (0-2) | 2 (1-3) | 0 (0-2) | Bacterial |
| COVID-19 | 1 | 73 | 0 | 1 (1-1) | 0 (0-0) | 0 (0-0) | 1 (1-1) | 0 (0-0) | 1 (1-1) | 0 (0-0) | Viral |
| Dengue | 4 | 8052 | 7 | 1 (1-3) | 0 (0-0) | 0 (0-0) | 0 (0-0) | 1 (0-3) | 1 (1-3) | 0 (0-14) | Viral |
| Diphtheria** | 3 | 6673 | 301 | 3 (2-6) | 0 (0-0) | 0 (0-0) | 3 (2-11) | 6 (3-6) | 9 (8-14) | 0 (0-1) | Bacterial |
| Hand Foot and Mouth | 1 | 300 | 0 | 14 (14-14) | 0 (0-0) |  |  |  |  | 0 (0-0) | Viral |
| Hepatitis E | 1 | 116 | 5 | 2 (2-2) | 1 (1-1) | 0 (0-0) | 1 (1-1) | 1 (1-1) | 2 (2-2) | 37 (37-37) | Viral |
| Influenza** | 1 | 54 | 0 | 8 (8-8) | 0 (0-0) | 0 (0-0) | 0 (0-0) | 7 (7-7) | 7 (7-7) | 7 (7-7) | Viral |
| Lassa fever | 3 | 51 | 18 | 5 (3-6) | 0 (0-5) | 0 (0-4) | 0 (0-1) | 0 (0-1) | 0 (0-1) | 0 (0-1) | Viral |
| Listeria | 1 | 1 | 1 | 7 (7-7) | 0 (0-0) | 0 (0-0) | 0 (0-0) | 4 (4-4) | 4 (4-4) | 0 (0-0) | Bacterial |
| Malaria | 1 | 35412 | 69 | 6 (6-6) | 45 (45-45) | 0 (0-0) | 0 (0-0) | 0 (0-0) | 0 (0-0) | 4 (4-4) | Parasitic |
| Marburg | 1 | 66 | 15 | 18 (18-18) | 0 (0-0) | 0 (0-0) | 0 (0-0) | 1 (1-1) | 1 (1-1) | 1 (1-1) | Viral |
| Measles** | 25 | 57782 | 360 | 4 (2-6) | 1 (0-3) | 0 (0-1) | 1 (0-5) | 2 (1-5) | 5 (3-8) | 6 (1-11) | Viral |
| Meningitis** | 1 | 3816 | 316 | 9 (9-9) | 103 (103-103) | 0 (0-0) | 8 (8-8) | 1 (1-1) | 9 (9-9) | 0 (0-0) | Bacterial (mostly) |
| Mpox** | 14 | 8478 | 46 | 5 (3-14) | 0 (0-0) | 0 (0-1) | 0 (0-1) | 1 (0-3) | 1 (1-3) | 4 (0-24) | Viral |
| Poliomyelitis** | 4 | 18 | 0 | 3 (2-18) | 12 (2-43) | 0 (0-0) | 8 (2-15) | 35 (23-58) | 47 (28-74) | 2 (2-81) | Viral |
| Rabies** | 1 | 20 | 3 | 12 (12-12) | 0 (0-0) | 1 (1-1) | 0 (0-0) | 1 (1-1) | 1 (1-1) | 2 (2-2) | Viral |
| Rift Valley Fever | 2 | 79 | 1 | 5 (3-6) | 0 (0-0) | 0 (0-0) | 2 (2-3) | 3 (2-4) | 5 (4-7) | 9 (4-13) | Viral |
| Chronic ulcers of unknown origin (“LADDE”) | 1 | 2945 | 0 | 0 (0-0) | 29 (29-29) | 31 (31-31) | 17 (17-17) | 7 (7-7) | 24 (24-24) | 122 (122-122) | Unknown |
| Pertussis ** | 1 | 50 | 0 | 4 (4-4) | 4 (4-4) | 0 (0-0) | 4 (4-4) | 4 (4-4) | 8 (8-8) | 0 (0-0) | Bacterial |
| Yellow fever** | 3 | 474 | 3 | 1 (1-4) | 0 (0-1) | 0 (0-0) | 1 (1-2) | 1 (1-5) | 4 (2-7) | 0 (0-0) | Viral |
| Overall | 83 | 228526 | 2646 | 4 (1-7) | 0 (0-2) | 0 (0-0) | 1 (0-2) | 2 (1-4) | 3 (1-8) | 1 (0-8) |  |

(*) 0 day means less than 24 hours ; (**) Vaccine-Preventable Diseases; (1) only 24 outbreaks were included in the calculation for measles; (2) only 82 outbreaks were included in the calculation of the indicators for the overall
